# Supplementary material for: Metabolic reprogramming dynamics in tumor spheroids: Insights from a multicellular, multiscale model
Source: PLoS Comput Biol. 2019 Jun 11;15(6):e1007053. doi: 10.1371/journal.pcbi.1007053 (PMC6588258; doi:10.1371/journal.pcbi.1007053)
Supplement: S1 Text — Supplemental Tables and Figures, along with detailed explanation of parameters listed in Table 1. (PDF) [file pcbi.1007053.s001.pdf]

Metabolic reprogramming dynamics in tumor spheroids: Insights from a multicellular, multiscale model

Mahua Roy[1], Stacey D. Finley [1]

1 Biomedical Engineering, Viterbi School of Engineering, University of Southern California, Los Angeles, California, USA

Supplementary Tables

Table A. Values of constants used in the model simulations

Quantity	Significance	Model values
1 pixel	Unit length of grid	4 μm
1 voxel	Unit surface area of grid	16 μm^2
1 voxel	Unit volume of grid	64 μm^3
NeighborOrder	Interaction Range	3
T_m	Internal fluctuation amplitude	50
α	Secretion rate of nutrients	0.5 fmol/voxel (0.0222 fmol/cell/sec)
Glucose in blood	Steady State Concentration	5 mM (0.32 fmol/voxel)
Oxygen in blood	Steady State Concentration	0.056 mM (0.003584 fmol/voxel)
Glutamine in blood	Steady State Concentration	1 mM (0.064 fmol/voxel)
Lactate in blood	Steady State Concentration	2 mM (0.128 fmol/voxel)
ϵ_{Glc}	Decay rate of glucose	1.562/mcs
ϵ_{Oxy}	Decay rate of oxygen	139.5089/mcs [1]
ϵ_{Gln}	Decay rate of glutamine	7.8125/mcs [2]
ϵ_{Lac}	Decay rate of lactate	3.9062/mcs [3]
D_{glc}	Diffusion constant of glucose	500 μm^2/s (11250 voxel/mcs))
D_{oxy}	Diffusion constant of oxygen	1820 μm^2/s (40950 voxel/mcs)) [1]
D_{gln}	Diffusion constant of glutamine	567 μm^2/s (12575.5 voxel/mcs))
D_{lac}	Diffusion constant of lactate	178 μm^2/s (4005 voxel/mcs)) [4]
λ	Chemotaxis coefficient	500

Table B. Fixed set of parameter values

Parameters	Significance	Values
V0	Target Volume	16
S0	Target Surface	16
λ_{V0}	Volume Constraint	15
λ_{S0}	Surface Constraint	5
$V0_{nec}$	Target Volume of a necrotic cell	4
$S0_{nec}$	Target Surface of a necrotic cell	4
λ_{NV0}	Volume Constraint of necrotic cell	10
λ_{NS0}	Surface Constraint of necrotic cell	5
EV0	Target Volume of an epithelial cell	40*V0
ES0	Target Surface of an epithelial cell	40*S0
λ_{EV0}	Volume Constraint of an epithelial cell	40*λ_{V0}
λ_{ES0}	Surface Constraint of an epithelial cell	40*λ_{S0}
kgts	surface = kgts*sqrt(volume)	4
Pvolmaxmit	Maximum volume of a proliferating cell	2*V0
Svolmaxmit	Maximum surface of a proliferating cell	2*S0
MaxDistance	Distance between COM of two epithelial cells	4.5
TargetDistance	Target Distance between two epithelial cells	4.0
$\lambda_{ecm-ecm}$	Distance constraint between ECM cells	60
$\lambda_{ecm-basal}$	Distance constraint between ECM and Basal cells	40
$\lambda_{basal-basal}$	Distance constraint between Basal cells	60
QCNeThr	QCancer to Necrotic transition threshold	2*PNeThr
PSNeThr	PStem to Necrotic transition threshold	4*PNeThr
QSNeThr	QStem to Necrotic transition threshold	8*PNeThr

Table C. Adhesion Coefficients for different cell types

Cell Types	Medium	PCancer	QCancer	PStem	QStem	Necrotic	Basal	ECM
**Medium**	0	10	10	10	10	10	2	2
**PCancer**	10	2	10	10	10	10	6	6
**QCancer**	10	10	2	10	10	10	8	8
**PStem**	10	10	10	2	10	10	6	6
**QStem**	10	10	10	10	2	10	8	8
**Necrotic**	20	10	10	10	10	2	20	20
**Basal**	2	6	8	6	8	20	10	16
**ECM**	2	6	8	6	8	20	16	10

Table D. Mean distribution of cells and volume fold change for different numbers of simulations after simulating 25 days of growth

Cell Types	50 simulations	100 simulations
**PCancer**	0.296 ± 0.114	0.271 ± 0.122
**QCancer**	0.034 ± 0.036	0.029 ± 0.034
**PStem**	0.177 ± 0.119	0.212 ± 0.138
**QStem**	0.033 ± 0.056	0.034 ± 0.052
**Necrotic**	0.461 ± 0.043	0.454 ± 0.042
**Volume fold change**	12.359 ± 1.675	12.476 ± 1.261

Supplementary Figures

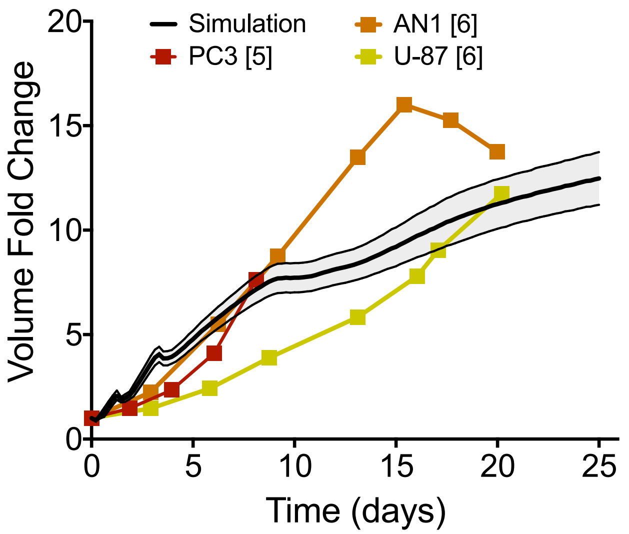

Figure A. Comparison of model predictions with experimental data. Model-simulated tumor growth profile using the optimized parameter set, initiating with the five different cell cluster configurations. Ten iterations for each case were simulated. The black solid line is the mean of all 50 simulations, and the shaded grey area represents the standard deviation of the simulations. Squares represent published experimental tumor spheroid data obtained from a prostate cancer cell line (PC3) [5] and two different glioma cell lines (AN1 and U-87) [6].

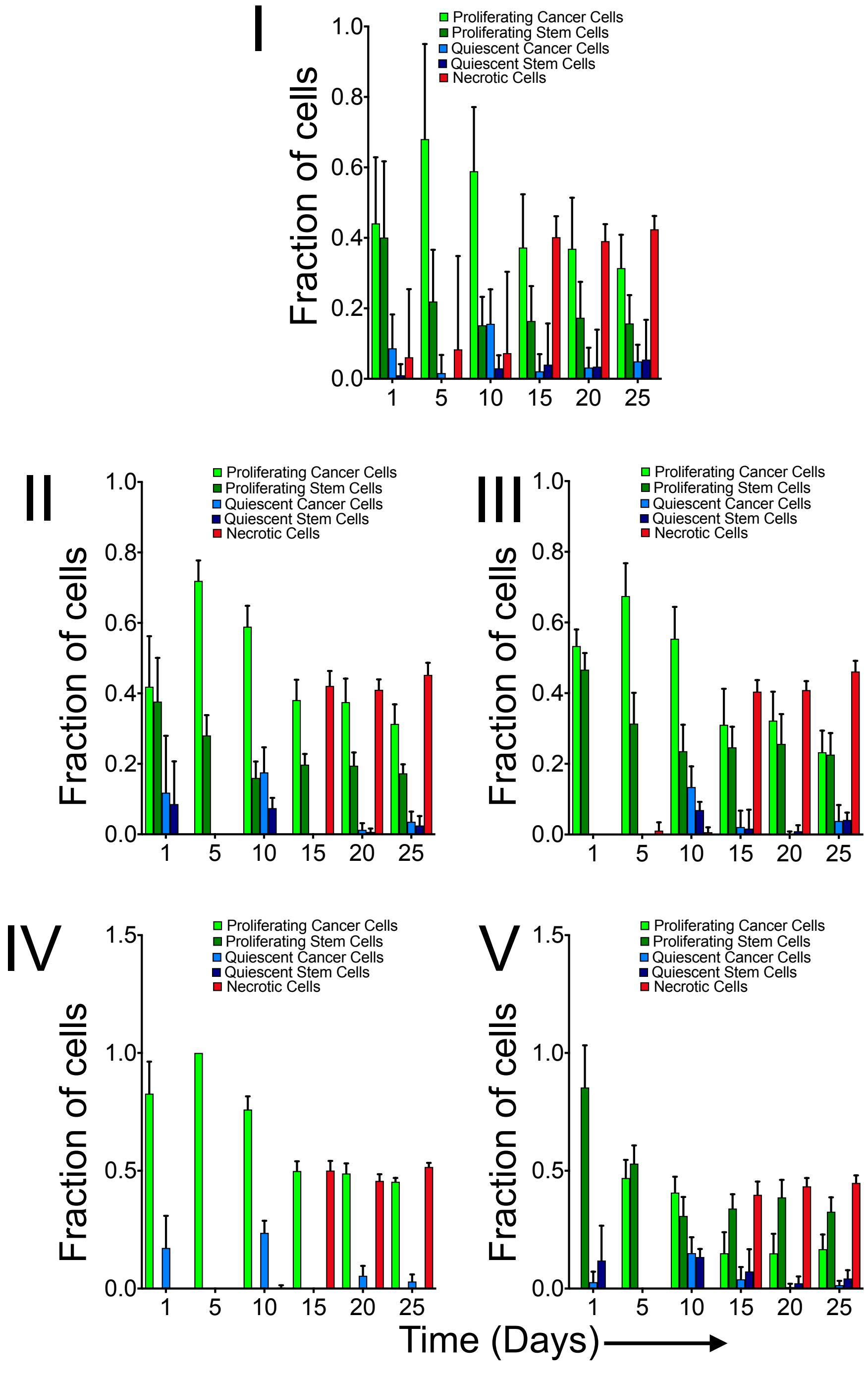

Figure B. Distribution of different cell types. The number of each cell type varies with time when the tumor is initiated with different configurations (see Figure 3(a)): (I) All four types of cells (PCancer, QCancer, PStem and QStem), (II) Only proliferating cell (PCancer and PStem), (III) Only quiescent cells (QCancer and QStem), (IV) Only proliferating cancer cell (PCancer) and (V) Only proliferating stem cells (PStem).

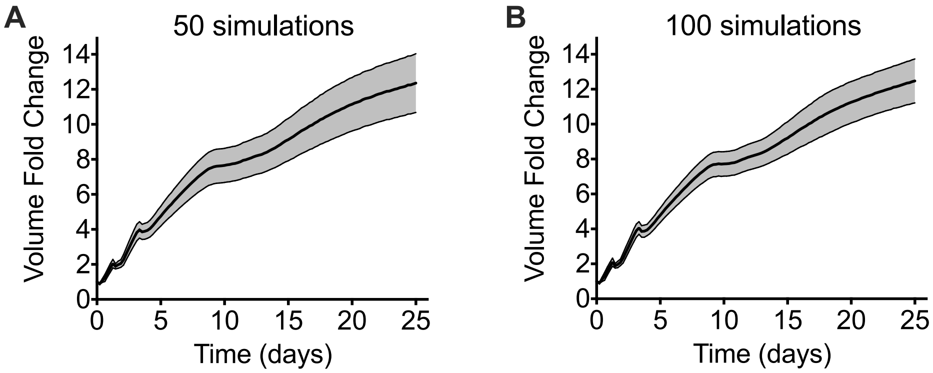

Figure C. Effect of additional simulations - Volume fold-change. Model-simulated tumor growth profile using the optimized parameter set, initiating with the five different cell cluster configurations. The black solid line is the mean of the simulations, and the shaded grey area represents the standard deviation of the simulations. A total of (a) 50 simulations (10 per case) or (b) 100 simulations (20 per case) were performed.

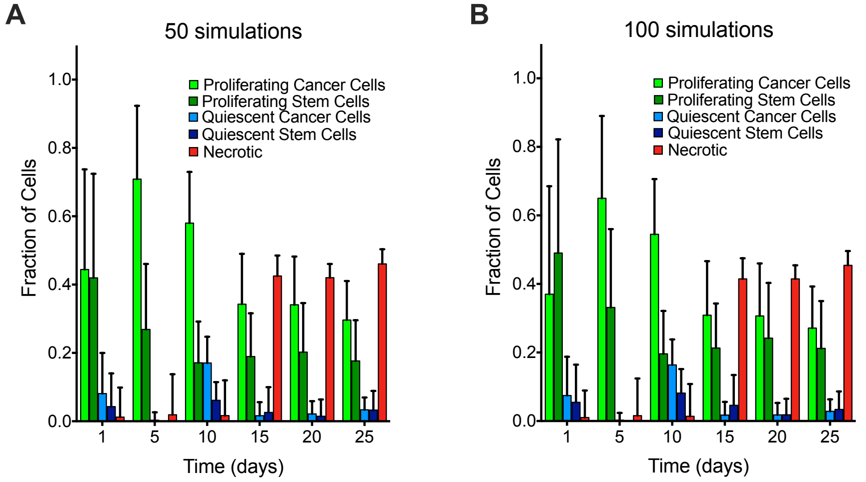

Figure D. Effect of additional simulations - Distribution of different cell types. The number of each cell type varies with time when the tumor is initiated with different configurations (see Figure 3(a)): (I) All four types of cells (PCancer, QCancer, PStem and QStem), (II) Only proliferating cell (PCancer and PStem), (III) Only quiescent cells (QCancer and QStem), (IV) Only proliferating cancer cell (PCancer) and (V) Only proliferating stem cells (PStem). A total of (a) 50 simulations (10 per case) or (b) 100 simulations (20 per case) were performed.

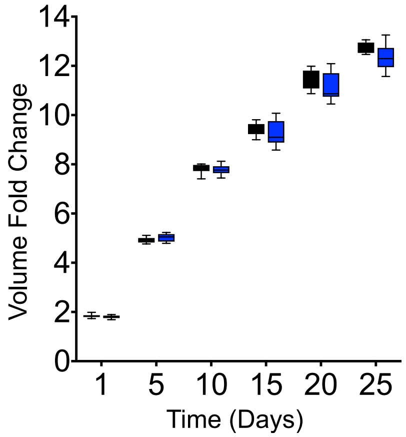

Figure E. Simulating different initial metabolite concentrations. Model simulations obtained when each cell is initiated with a slight variation in the intracellular conditions, eventually generating greater inter-tumor heterogeneity as the tumor grows. The figure depicts the time profile of tumor growth for 10 iterations of the control case where each simulation starts with same intracellular metabolite concentrations for each cell in the initial cluster (black) and 10 iterations where each cell has a different set of initial conditions (blue). The error bars represent the standard deviation.

Description of parameters given in Table 1 of the main text

1. *incvol*: The rate at which cells increase their volume to proliferate, a property of only proliferating cells (PCancer and PStem). The higher the rate, the greater the increase in the cell's volume, leading to a higher rate of proliferation.
2. *decvol*: The rate at which necrotic cells decrease their volume, mimicking apoptosis.
3. *PGrThr*: The threshold value for the total concentration of ATP, intracellular glucose and glutamine needed for a proliferating cell to increase its volume.
4. *SGrThr*: The threshold value for the total concentration of ATP, intracellular glucose and glutamine needed for a stem cell to increase its volume.
5. *StressIncrement*: The value by which the Stress attribute is increased when the required number of neighbors (N) surround a cell and exert compressive stress.
6. *StressThr*: The threshold value of the Stress attribute that the cells have to cross to transition into a necrotic cell.
7. N: The number of neighbors that a cell has, which influences the accumulation of Stress.
8. V_{atpmax}: A parameter influencing how cell attributes depend on ATP.
9. *atpD*: Threshold value below which the Starvation attribute is incremented for the cell, leading to necrosis and above which Health is acquired, leading to the conversion of a quiescent cell to a proliferating cell.
10. $PNeThr$: This is the threshold for a proliferating cell to transition into necrotic state due to a lack of ATP.
11. $PNeThr_{acidosis}$: This is the threshold for a proliferating cell to transition into necrotic state due to excessive exposure to an acidic environment.
12. $Total_{time}$: The total time for which the cells have to be exposed to a low ($Neg_{concATP}$) or high ($Pos_{concATP}$) concentration of ATP in order to transition to a necrotic or proliferating cell.
13. $Neg_{concATP}$: The low concentration of ATP used in the calculation of *PNeThr*. A low value of $Neg_{concATP}$ leads to low value of *PNeThr* and an easier transition of a proliferating cell to a necrotic cell. Alternatively, a high value of $Neg_{concATP}$ leads to high value of *PNeThr* respectively and an harder transition of a proliferating cell to a necrotic cell.
14. $Pos_{concATP}$: Total amount of intracellular ATP, glucose and glutamine used in the calculation of *QCPThr* and *QSSThr*, which affects the transition from a quiescent cell to a proliferating cell.
15. V_{lacmax}: A parameter for attribute factor calculation dependent on lactate.
16. *LacDeath*: Threshold value of lactate concentration, above which the Starvation attribute is incremented for the cell, leading to necrosis.
17. $Pos_{concLac}$: The high concentration of lactate used in the calculation of $PNeThr_{acidosis}$.

18. $Total_{timelac}$: This is the total time for which the cells have to exposed to high ($Pos_{concLac}$) concentration of lactate to transition to necrotic state due to acidosis.

19. *maxdiv*: Maximum number of divisions the cell can have before senescence sets in and the cell transitions to a necrotic cell.

20. *probstem*: The probability of a proliferating stem cell (PStem) to divide into a quiescent cancer (QCancer) or quiescent stem (QStem) cell.

21. C: This measures the amount of contribution of nutrients towards the accumulation of Health. Higher C values leads to faster accumulation of Health and an easier transition from a quiescent cell to a proliferating cell.

22. a: The Hill coefficient representing the cooperativity between nutrients responsible for the growth and transition of a cell.

References

1. Robertson-Tessi M, Gillies RJ, Gatenby RA, Anderson AR. Impact of metabolic heterogeneity on tumor growth, invasion, and treatment outcomes. Cancer research. 2015;75(8):1567–1579.

2. Mathews EH, Stander BA, Joubert AM, Liebenberg L. Tumor cell culture survival following glucose and glutamine deprivation at typical physiological concentrations. Nutrition. 2014;30(2):218–227.

3. Kennedy KM, Scarbrough PM, Ribeiro A, Richardson R, Yuan H, Sonveaux P, et al. Catabolism of exogenous lactate reveals it as a legitimate metabolic substrate in breast cancer. PloS one. 2013;8(9):e75154.

4. Kasinskas RW, Venkatasubramanian R, Forbes NS. Rapid uptake of glucose and lactate, and not hypoxia, induces apoptosis in three-dimensional tumor tissue culture. Integrative Biology. 2014;6(4):399–410.

5. Jones DT, Valli A, Haider S, Zhang Q, Smethurst EA, Schug ZT, et al. 3D Growth of Cancer Cells Elicits Sensitivity to Kinase Inhibitors but Not Lipid Metabolism Modifiers. Molecular cancer therapeutics. 2019;18(2):376–388.

6. Tysnes BB, Maurer HR, Porwol T, Probst B, Bjerkvig R, Hoover F. Bromelain reversibly inhibits invasive properties of glioma cells. Neoplasia. 2001;3(6):469–479.
