# Supplementary material for: Metabolic reprogramming dynamics in tumor spheroids: Insights from a multicellular, multiscale model
Source: PLoS Comput Biol. 2019 Jun 11;15(6):e1007053. doi: 10.1371/journal.pcbi.1007053 (PMC6588258; doi:10.1371/journal.pcbi.1007053)
Supplement: S3 Text — This provides snippets of code from CC3D to demonstrate how the model is implemented. (PDF) [file pcbi.1007053.s003.pdf]

Metabolic reprogramming dynamics in tumor spheroids: Insights from a multicellular, multiscale model

Mahua Roy[1], Stacey D. Finley[1]

[1] Biomedical Engineering, Viterbi School of Engineering, University of Southern California, Los Angeles, California, USA

This file describes the CC3D simulation implementation of the modeling framework presented in the main text.

1. **Cell Types:** The specifications of the different cell types are as follows:

```
<Plugin Name="CellType">


      <CellType TypeId="0" TypeName="Medium"/>
      <CellType TypeId="1" TypeName="PCancer"/>
      <CellType TypeId="2" TypeName="QCancer"/>
      <CellType TypeId="3" TypeName="PStem"/>
      <CellType TypeId="4" TypeName="QStem"/>
      <CellType TypeId="5" TypeName="Necrotic"/>
      <CellType TypeId="6" TypeName="Basal"/>
      <CellType TypeId="7" TypeName="ECM"/>

   </Plugin>
```

2. **Cell Parameters:** These are the specific parameters in the model simulations. They have also been tabulated in the supplementary file "S1 Text".

    1) V0 = tumor cell target volume in voxel
    2) S0 = tumor cell target surface in voxel
    3) V0_nec = tumor cell volume of necrotic cells
    4) S0_nec = tumor cell surface of necrotic cells
    5) LBD_V0 = lambda value for volume
    6) LBD_S0 = lambda value for surface
    7) LBD_NV0 = lambda value for volume necrotic
    8) LBD_NS0 = lambda value for surface necrotic
    9) EV0 = epithelial cell target volume
    10) ES0 = epithelial cell target surface
    11) LBD_EV0 = lambda value for epithelial cell volume
    12) LBD_ES0 = lambda value for epithelial cell surface
    13) kgts = factor multiplying for surface (PLOS ONE 2015)
    14) Pvolmaxmit = tumor cell mitosis volume (PCANCER)
    15) Svolmaxmit = stem cell mitosis volume (PSTEM)
    16) a = the Hill coefficient
    17) incvol = growth rate of tumor cells in pixels/MCS
    18) decvol = decreasing rate of tumor cells in pixels/MCS
    19) PGrThr = concentration above which proliferating cell grows
    20) SGrThr = concentration above which stem cell grows
    21) StressIncrement = Stress increment due to neighbors
    22) PUgMax = Proliferating cell - maximum uptake rate
    23) QUgMax = 0.75*PUgMax = Quiescent cell - maximum uptake rate
    24) atpK = Michaelis-Menten constant for ATP concentration
    25) atpD = ATP concentration below which damage accumulates:5mM
        = 0.32 fmol/vox
    26) Total_time = Time for which the cell is exposed for minimum or maximum nutrients
    27) Neg_concATP = Lowest minimum possible concentration
    28) PNeThr = (Total_time) *(PUgMax*(Neg_concATP**a/(Neg_concATP**a + atpK**a)))
        = total damage at which proliferating -> necrotic
    29) QCNeThr = 2*PNeThr = total damage at which: quiescent -> necrotic
    30) SNeThr = 4*PNeThr
        = total damage at which: stem -> necrotic
    31) QSNeThr = 8*PNeThr
        = total damage at which: quiescent -> necrotic
    32) Pos_concATP = Concentration of ATP which helps acquire health
    33) QCPThr = (Total_time) *(QUgMax*(Pos_concATP**a/(Pos_concATP**a + atpK**a))
        = total happiness at which: quiescent -> proliferating
    34) QSSThr = QCPThr = total happiness at which: QS -> S
    35) PLUgMax = 15.393 = Proliferating cell - maximum uptake rate
    36) QLUgMax = 0.75*PLUgMax = Quiescent cell - maximum uptake rate
    37) lacK = Michaelis-Menten constant for ATP concentration
    38) LacDeath = Threshold at which the necrosis sets in due to acidity from Lactate concentration
    39) Pos_concLac = concentration of Lactate causes death
    40) Total_time_lac = Total time for which the cell is exposed to Lactate

41) PNe_AThr = (Total_time_lac) *(PLUgMax*(Pos_concLac**a/(Pos_concLac**a + lacK**a)))
= total damage at which: proliferating -> necrotic
42) QCNe_AThr = 2*PNe_AThr = total damage at which: quiescent -> necrotic
43) SNe_AThr = 4*PNe_AThr = total damage at which: stem -> necrotic
44) QSNe_AThr = 8*PNe_AThr = total damage at which: quiescent -> necrotic
45) maxdiv = maximum number of cell divisions
46) probstem = probability of new stem cell after division
47) StressThr = Cell Stress threshold
48) K = Coefficient for cooperativity due to multiple nutrients leading to growth
49) Neigh = The number of neighbors above which the cells begin to accumulate stress

3. **Intracellular Metabolic Network:** The metabolic network is represented as a reaction network of ordinary differential equations. These differential equations are solved for each cell using an SBML file (**S3_diffusion.xml**) attached to each live cell (`PCancer`,`QCancer`,`PStem`,`QStem`). The concentration of the extracellular nutrients at the center of mass of each cell on the grid is calculated and is given as initial conditions into the SBML file. The model is then solved for each MCS (corresponding to 6 minutes).

```
def start(self):
        options={'relative':1e-09,'absolute':1e-12}
        self.setSBMLGlobalOptions(options)
        modelFile = './Simulation/S3_diffusion.xml'
        stepSize = 6

self.addSBMLToCellTypes(_modelFile=modelFile,_modelName='PC_ODE',
_types=[self.PCANCER,              self.QCANCER,              self.PSTEM,
self.QSTEM],stepSize=stepSize)

 self.setSBMLValue(_modelName='PC_ODE',_valueName='Glu_out',
_value=Glu_out,_cell=cell)

self.setSBMLValue(_modelName='PC_ODE',_valueName='Glutamine_out,
_value=Glutamine_out,_cell=cell)

self.setSBMLValue(_modelName='PC_ODE',_valueName='O2e',_value=O2e,_cell
=cell)

self.setSBMLValue(_modelName='PC_ODE',_valueName='Lac_out',_value=Lac_o
ut,_cell=cell)

 if mcs > 1:
               self.timestepSBML()
```

4. **Cell Volume and Surface Constraints:** The tumor growth steppable sets the target volume (`cell.targetVolume`), the lambda volume (which influences the volume constraints, `cell.lambdaVolume`), as well as the surface constraints for the various cells including the necrotic cells. The cell attributes are initiated at time zero.

```
for cell in self.cellListByType(self.PCANCER, self.QCANCER, self.PSTEM, self.QSTEM):
            cell.targetVolume=V0
            cell.lambdaVolume=LBD_V0
            cell.targetSurface=S0
            cell.lambdaSurface=LBD_S0
```

```
for cell in self.cellListByType(self.NECROTIC):
            cell.targetVolume=V0_nec
            cell.lambdaVolume=LBD_NV0
            cell.targetSurface=S0_nec
            cell.lambdaSurface=LBD_NS0
```

```
cellDict=CompuCell.getPyAttrib(cell)

            cellDict["Counter"] = 0
            cellDict["Health"] = 0
            cellDict["Starvation"] = 0
            cellDict["Acidity"] = 0
            cellDict["Stress"] = 0
```

5. **Cell Growth and Death:** After every 50th MCS, the proliferating cells are examined for the amount of glucose, glutamine and ATP. If the total sum of these nutrients (`conc_gr`) is greater than the growth threshold (`PGrThr` for self.PCANCER, `SGrThr` for self.PSTEM), the cell's target volume increases by the difference, (`conc_gr - PGrThr`, `conc_gr - SGrThr`). This volume increases for proliferating cells only.

```
if cell.type == self.PCANCER:
             conc_gr =(0.32/5)*(cellDict["Glu_in"]+cellDict["Glutamine_in"]
                      + cellDict["ATP"])
            concentration1 = max(0,conc_gr - PGrThr)
            cell.targetVolume+=incvol*(concentration1)
            cell.targetSurface=kgts*sqrt(cell.targetVolume)
if cell.type == self.PSTEM:
            conc_gr=(0.32/5)*(cellDict["Glu_in"]+cellDict["Glutamine_in"]
                     + cellDict["ATP"])
            concentration2 = max(0,conc_gr - SGrThr)
            cell.targetVolume+=incvol*(concentration2)
            cell.targetSurface=kgts*sqrt(cell.targetVolume)
```

The necrotic cells, on the other hand, are examined to see if they are on the periphery of the tumor. In this case, the cell's target volume continues to diminish by an amount equal to `decvol`. If the necrotic cell is not on the tumor periphery, its target volume shrinks to ¼ of the original volume. The target volume of necrotic cells is assigned based on previous work (4).

```
if cell.type == self.NECROTIC :
                  cellNeighborList=self.getCellNeighborDataList(cell)

                  neighbor_count_by_type=
cellNeighborList.neighborCountByType()

               if neighbor_count_by_type[0] >= 1:
                  cell.targetVolume-= min(decvol,cell.targetVolume)
               else:
                  cell.targetVolume-= 0
                  cell.targetSurface=kgts*sqrt(cell.targetVolume)
```

6. **Cell State Transition**: The cell's transition from one state to another depends on four attributes, which are initiated at the beginning of the simulations:

```
cellDict["Health"] = 0
cellDict["Starvation"] = 0
cellDict["Acidity"] = 0
cellDict["Stress"] = 0
```

   a. *PCancer and PStem cells*: If the cell's attributes of `Starvation`, `Acidity`, `Stress` cross their respective thresholds (`PNeThr`, `PNe_AThr`, `StressThr`) they become necrotic. Upon cell death, it releases its intracellular lactate into the environment at its respective coordinates (`LactateField.set`). Additionally, the cell attribute of `Health` becomes zero.

```
if cell.type == self.PCANCER:
   if  (cellDict["Starvation"]  >  PNeThr  or  cellDict["Acidity"]  >
PNe_AThr or cellDict["Stress"] > StressThr):
   LactateField.set(pt,(0.32/5)*(XLac + XLacout))
   cellDict["exLactate_now"] = LactateField.get(pt)
   cell.type=self.NECROTIC
   cellDict["Health"]=0

if cell.type == self.PSTEM:
   if  (cellDict["Starvation"]  >  SNeThr  or  cellDict["Acidity"]  >
SNe_AThr or cellDict["Stress"] > StressThr):
  LactateField.set(pt,(0.32/5)*(XLac + XLacout))
  cellDict["exLactate_now"] = LactateField.get(pt)
  cell.type=self.NECROTIC
  cellDict["Health"]=0
```

   b. *QCancer and QStem cells*: Quiescent cells can either become Necrotic or they can become proliferating Cancer and Stem cells. Similar to *PCancer* and *PStem* cells, *QCancer* and *QStem* have their respective damage threshold (tabulated in Table 2, Supplementary File S1). When the given threshold value is reached, the cell transitions into the Necrotic state, as described above.

```
if cell.type==self.QCANCER:
   if  (cellDict["Starvation"]  >  QCNeThr  or  cellDict["Acidity"]  >
QCNe_AThr or cellDict["Stress"] > StressThr):
   LactateField.set(pt,(0.32/5)*(XLac + XLacout))
   cellDict["exLactate_now"] = LactateField.get(pt)
   cell.type=self.NECROTIC
   cellDict["Health"]=0
```

   However, both *QCancer* and *QStem* cells can gain `Health`, and if this attribute reaches the threshold value (`QCPThr` or `QSPThr`), the cell transitions into a proliferating cell.

```
if cellDict["Health"] > QCPThr:
                               cell.type=self.PCANCER
                               cellDict["Health"]=0
if cellDict["Health"] > QCSThr:
                               cell.type=self.PSTEM
                               cellDict["Health"]=0
```

7. **Calculating the Cell Attributes**:
For all cell types, the cell's dictionary for attributes is initiated at 0.

```
for  cell  in  self.cellListByType(self.PCANCER,  self.QCANCER,  self.PSTEM,
self.QSTEM, self.NECROTIC):
            cellDict=CompuCell.getPyAttrib(cell)

            cellDict["Starvation"]=0
            cellDict["Health"]=0
            cellDict["Acidity"]=0
            cellDict["Stress"]=0
```

The attributes are calculated by a Michaelis Menten equation

```
def MM(self,x,m,k,coeff):

        return (m*(x**coeff/(x**coeff + k**coeff)))
```

The cell's damage ("`Starvation`") is increased whenever the concentration of ATP falls below the ATP death threshold (`atpD`)

```
if conc_atp < atpD :
   cellDict["Starv"]+=abs(self.MM(conc_atp,PUgMax,atpK,a)-
self.MM(atpD,PUgMax,atpK,a))
```

Similarly, the attribute that corresponds to cell's death due to acidity ("`Acidity`") is increased when the extracellular lactate concentration around the cell becomes greater than the Lactate Death threshold.

```
elif conc_lacout > LacDeath:
     cellDict["Acidity"]+=abs(self.MM(conc_lacout,PLUgMax,lacK,a)-
self.MM(LacDeath,PLUgMax,lacK,a))
```

Otherwise, there is sufficient ATP, and the cell is able to increase its health and grow

```
else:

cellDict["Health"]+=exp(K)*(self.MM(conc_gr,PUgMax,atpK,4))
```

Nutrient concentrations influence starvation (intracellular ATP; `conc_atp`), acidity (extracellular lactate; `conc_lacout`) and growth (the sum of intracellular glucose, glutamine, and ATP; `conc_gr`). These concentrations are calculated as follows:

```
conc_atp = (0.32/5)*cellDict["ATP"]
conc_lacout =  (0.32/5)*cellDict["exLactate_now"]
conc_gr =(0.32/5)*(cellDict["Glu_in"]+cellDict["Glutamine_in"]+cellDict["ATP"])
```

8. **Cell Mitosis**: A cell divides when its volume exceeds the maximum volume values required for division (`Pvolmaxmit, Svolmaxmit`). Cells divide along a random orientation, i.e., there is no particular axial direction for their division.

```
for cell in self.cellList:


     if((cell.type==self.PCANCER      or      cell.type==self.QCANCER)      and
cell.volume>Pvolmaxmit) or ((cell.type==self.PSTEM or cell.type==self.QSTEM)
and cell.volume>Svolmaxmit):



     cells_to_divide.append(cell)


for cell in cells_to_divide:
        self.divideCellRandomOrientation(cell)
```

9. **Extracellular nutrients: Secretion and Diffusion**: The extracellular space is comprised of the stromal compartment (modeled as a continuous medium) and epithelial cells (modeled as discrete cells). The extracellular compartment, which includes the stromal and epithelial layers, secretes the nutrients (glucose, glutamine, oxygen and lactate) that contribute to the tumor cells' growth and death. Glucose, glutamine and oxygen are secreted by both Stromal cells ("Medium") and extracellular matrix ("Basal" and "ECM") and diffused in the extracellular space. Lactate is only secreted by the Stromal cells and diffused similarly.

```
Name="Glucose">
<FieldName>Glucose</FieldName>
<DiffusionConstant>11250.0</DiffusionConstant>
<DecayConstant>1.562</DecayConstant>
<InitialConcentrationExpression>0.32</InitialConcentrationExpression>
<SecretionData>
<Uptake Type="QStem"/>   # These are the cells taking up the nutrients
<Uptake Type="PStem"/>
<Uptake Type="QCancer"/>
<Uptake Type="PCancer"/>
<Secretion Type="Medium">0.5</Secretion>
<Secretion Type="ECM">0.5</Secretion>
<Secretion Type="Basal">0.5</Secretion>
</SecretionData>
```

```
Name="Lactate">
<FieldName>Lactate</FieldName>
<DiffusionConstant>4005.0</DiffusionConstant>
<DecayConstant>3.9062</DecayConstant>
<InitialConcentrationExpression>0.128</InitialConcentrationExpression>
<SecretionData>
<Uptake Type="QStem"/>
<Uptake Type="PStem"/>
<Uptake Type="QCancer"/>
<Uptake Type="PCancer"/>
<Secretion Type="Medium">0.5</Secretion>
</SecretionData>
```
