# Supplementary figures and images for: Metabolic reprogramming dynamics in tumor spheroids: Insights from a multicellular, multiscale model
Source: PLoS Comput Biol. 2019 Jun 11;15(6):e1007053. doi: 10.1371/journal.pcbi.1007053 (PMC6588258; doi:10.1371/journal.pcbi.1007053)

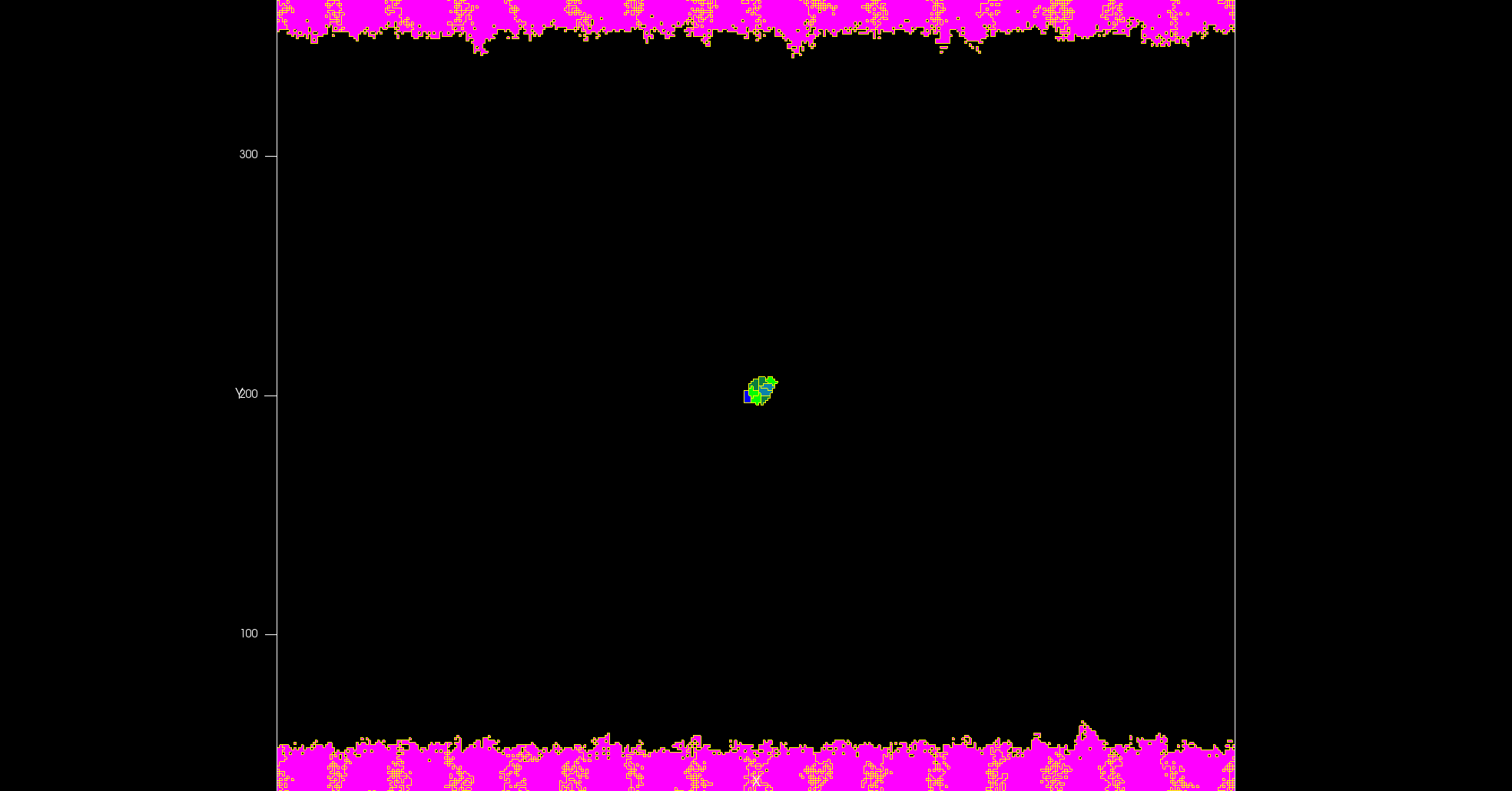

Supplement: S1 Movie — (GIF) [file pcbi.1007053.s005.gif]
